# Genome-wide Identification, Classification, Molecular Evolution and Expression Analysis of Malate Dehydrogenases in Apple

**DOI:** 10.3390/ijms19113312

**Published:** 2018-10-24

**Authors:** Baiquan Ma, Yangyang Yuan, Meng Gao, Libo Xing, Cuiying Li, Mingjun Li, Fengwang Ma

**Affiliations:** State Key Laboratory of Crop Stress Biology for Arid Areas/Shaanxi Key Laboratory of Apple, College of Horticulture, Northwest A&F University, Yangling 712100, Shaanxi, China; bqma87@nwsuaf.edu.cn (B.M); yy.yuan@nwsuaf.edu.cn (Y.Y.); gaomeng086630@gmail.com (M.G.); libo_xing@nwsuaf.edu.cn (L.X.); lcy1262@sina.com (C.L.)

**Keywords:** apple, malate dehydrogenase, evolutionary pattern, positive selection, expression profile

## Abstract

Malate dehydrogenase plays crucial roles in energy homeostasis, plant development and cold and salt tolerance, as it mediates the reversible conversion of malate to oxaloacetate. However, the evolutionary pattern of *MDH* genes in apple remains elusive. In this study, a total of 20 *MDH* genes were identified from the “Golden Delicious” apple draft genome. We revealed the physiological and biochemical properties, gene structure, and conserved motifs of *MdMDH* genes. Chromosomal localization and *Ka*/*Ks* ratio analysis of *MdMDH* genes revealed different selective pressures acted on duplicated *MdMDH* genes. Exploration of the phylogenetic relationships revealed six clades and similar frequencies between old and recent duplications, and significant differences in the evolutionary rates of the *MDH* gene family were observed. One *MdMDH* gene, MDP0000807458, which was highly expressed during apple fruit development and flower bud differentiation, was under positive selection. Thus, we speculated that MDP0000807458 is a likely candidate gene involved in regulation of flower bud differentiation and organic acid metabolism in apple fruits. This study provides a foundation for improved understanding of the molecular evolution of *MdMDH* genes and further facilitates the functional analysis of MDP0000807458 to unravel its exact role in flower bud differentiation and organic acid metabolism.

## 1. Introduction

Malate dehydrogenase (*MDH*) is ubiquitous in plants, animals and microorganisms and catalyzes the reversible conversion of malate to oxaloacetate via NAD^+^ or NADP^+^ as a coenzyme, depending on the *MDH* isoform [1]. NADP-dependent *MDH* exists in the chloroplast, whereas NAD-dependent *MDH*s exist in the cytosol, plastids, mitochondria, peroxisomes and other microbodies. In general, all plant *MDH* isoforms are encoded in the cell nucleus, and are stable as homodimers that have similar coenzyme binding sites, active sites and quaternary structures. Two distinct domains, an NAD/NADP-binding domain and a substrate-binding domain, are present within each subunit, and the active site of MDH proteins is located in a cleft between the two domains [2,3].

*MDH* is a well-studied enzyme, and two predominant *MDH* forms have been discovered in most plant species: mitochondrial *MDH* and cytoplasmic *MDH*. Mitochondrial *MDH*s are involved in the tricarboxylic acid (TCA) cycle, while cytoplasmic *MDH*s function in both acid metabolisms within plant tissues and carbon dioxide fixation in C4 plants [4,5]. To date, the number of *MDH* gene members in different plants is highly diverse, and they have roles in a variety of physiological processes. In the *Arabidopsis* genome, nine putative *MDH* isoforms have been identified, including eight NAD-*MDH*s and one NADP-*MDH* [6]. Functional analyses have revealed that, in *Arabidopsis*, the plastid-localized NAD-*MDH* plays crucial roles in energy homeostasis, embryo development and heterotrophic metabolism [6,7]; however, two mitochondrial *MDH*s, m*MDH*1 and m*MDH*2, control the seed maturation and postgermination growth [8]. The *MDH* isozymes have been studied more intensively in maize than in other plant species [9]. In maize, *NADP-MDH* was proven to be a plastidic *MDH* gene, and its overexpression can increase salt stress tolerance [5]. Similarly, an *MDH* gene whose product is cytosolic has been identified; in the apple genome, this gene product can enhance tolerance to cold and salt stress [10,11]. Thus, during the evolutionary process of different species, *MDH* genes have undergone functional differentiation; it is therefore critical to evaluate the evolutionary relationships of *MDH* genes between different species.

Apple (*Malus* × *domestica* Borkh.) is one of the most popular fruits that are grown in the temperate regions worldwide, and its draft genome sequences have been released [12]. Apple belongs to the genus *Malus* in the family Rosaceae and has an autopolyploidy origin. During apple domestication, genome-wide duplication (GWD) played an important role in the transition from 9 ancestral chromosomes to 17 chromosomes of the Pyreae [12]. Gene duplication, which can arise from GWD, segmental duplication, random duplication or polyploidization, is an important influencing factor of plant morphogenetic evolution and a driving force for the recruitment of genes in plants. After duplication, genes can be nonfunctional (gene death), neofunctional (having a novel function) or subfunctional (having a part of the original function) [13]. In general, most duplicated genes exhibit a rapid divergence in expression [14] and accumulate deleterious mutations, after which they become nonfunctional. In contrast, a portion of duplicated genes that are under positive selection are eventually retained and evolve a novel function [13,15]. Thus, the evolutionary divergence of duplicated genes during the plant speciation process should be estimated.

In this study, the draft genome of the diploid apple cultivar “Golden Delicious” was first used to identify the *MDH* gene family in apple at genome-wide level. A total of 20 *MdMDH* genes were identified in the apple draft genome, and their physiological and biochemical properties, chromosomal localization, level of gene duplication, evolutionary rate, and selective pattern were estimated. In addition, the expression profiles of 20 *MdMDH* genes in different apple tissues are investigated, and we estimated the expression patterns of 20 *MdMDH* genes during apple flower bud differentiation and in fruits at different developmental stages. Our findings shed light on the molecular properties and evolutionary patterns of the *MdMDH* gene family and provide a theoretical basis for future in-depth elucidation of the biological functions of *MDH* genes during apple flower bud differentiation and fruit development.

## 2. Results

### 2.1. Identification and Characterization of 20 *MDH* Genes in Apple

In this study, a total of 20 putative *MDH* genes in the draft genome of the diploid apple cultivar “Golden Delicious” were identified [12], and their corresponding sequences are shown in Table S2. Furthermore, the physiological and biochemical analyses of the MdMDH proteins were characterized using ProtParam (https://web.expasy.org/protparam/), and the results are shown in Table 1. The length of the MdMDH proteins ranged from 134 (MDP0000807458) to 946 (MDP0000295823) amino acids, with predicted molecular weights of 14.48–103.76 kDa. The grand average of hydropathicity (GRAVY) of the MdMDH proteins ranged from −0.085 (MDP0000295823) to 0.231 (MDP0000277049), and the theoretical isoelectric point (pI) ranged from 5.99 (MDP0000272403) to 9.27 (MDP0000807458). In addition, the instability index of MdMDH proteins ranged from 24.2 (MDP0000277049) to 46.76 (MDP0000295823), and the aliphatic index ranged from 88.08 (MDP0000141199) to 105.5 (MDP0000191302).

**Figure 1:**
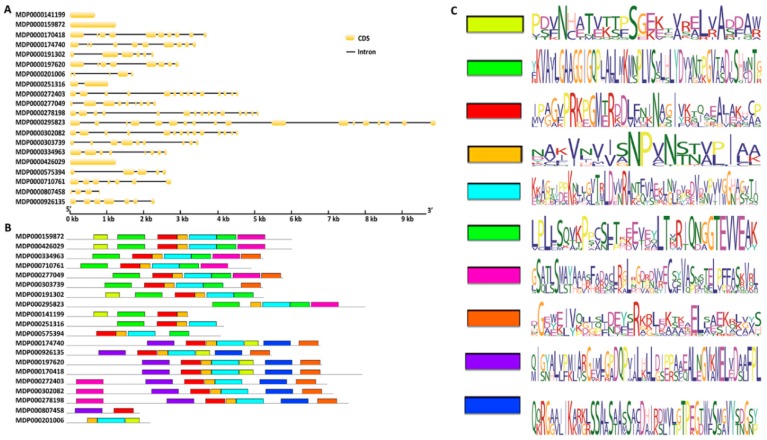
The genomic structure and conserved motifs of *MDH* genes in apple: (**A**) the exon–intron structure of apple *MDH* genes, where yellow indicates exons and black line indicates introns; (**B**) the distribution of conserved motifs in MdMDH proteins, where the conserved motifs are indicated by colored boxes; and (**C**) sequences of the 10 conserved motifs in MdMDH proteins identified in this study.

To better understand the genomic structure of the *MdMDH* genes, exon–intron diagrams of the *MdMDH* genes were generated according to their coding and genomic sequences (Figure 1A). The results showed that the exon number of each *MdMDH* gene ranged dramatically from 1 to 18 exons. Three *MdMDH* genes, MDP0000141199, MDP0000159872 and MDP0000426029, contained one exon, while one *MdMDH* gene, MDP0000295823, contained 18 exons. This finding suggests that both exon gain and loss have occurred in the *MdMDH* gene family during apple speciation. To discover the conserved motifs of *MdMDH* genes more systematically, the online MEME server was used to estimate the distribution of conserved motifs. The results showed that 10 putative conserved motifs were identified in the *MdMDH* gene family (Figure 1B), and the length of the conserved motifs ranged from 19 to 50 amino acids (Figure 1C). The number of conserved motifs within each *MdMDH* gene ranged from 2 to 7. The majority of the *MdMDH* genes had 4–7 conserved motifs; however, MDP0000807458 contained two conserved motifs, and MDP0000201006 contained three conserved motifs. Thus, the *MdMDH* genes clearly exhibited extreme divergence during the evolutionary process.

### 2.2. Chromosomal Localization and Duplication of MdMDH Genes

**Figure 2:**
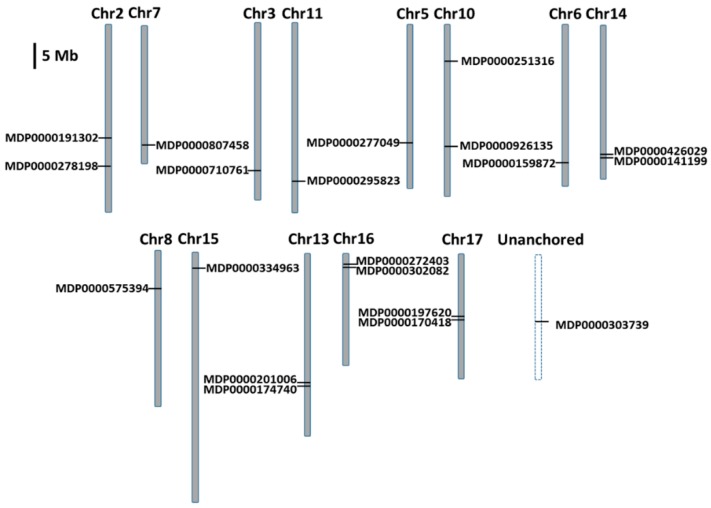
Chromosomal location of the apple *MDH* genes; the dashed line indicates the anchored sequences.

The genomic distribution of the 20 *MdMDH* genes is shown in Figure 2. Of these genes, 17 were identified on six homologous pairs of chromosomes (2–7, 3–11, 5–10, 6–14, 8–15 and 13–16), two were identified on chromosome 17, and one was identified among the unanchored sequences. In addition, whole-genome duplication (WGD) and segmental duplication occurred during the process of apple domestication and played an important role in the expansion of the apple gene family, as this process allows the retention of numerous duplicated genes within the apple genome [12,16]. To determine whether an *MdMDH* gene is under different selective pressures and exhibits evolutionary rates that differ from those of its duplicated genes, we estimated the evolutionary rates and selective pressure according to the *Ka* (nonsynonymous), *Ks* (synonymous) and *Ka*/*Ks* ratio (ω). In general, ω > 1 supports the hypothesis of positive selection, ω < 1 indicates purifying selection, and ω = 1 indicates neutral evolution. Thus, we investigated the selective pressures that act on duplicated *MdMDH* genes; the ω values of each duplicated gene pair are shown in Table S3. The ω value of the majority of the *MdMDH* gene pairs was less than 1, indicating that their evolution is under strong purifying selection. Interestingly, positive selection was detected in one pair of paralogs (MDP0000303739 and MDP0000191302), with a ratio of 1.15, and neutral selection was detected in one pair of paralogs (MDP0000807458 and MDP0000159872), with a ratio of 1.00.

### 2.3. Phylogenetic Analysis, Gene Duplication and Loss of MDH Genes in Four Rosaceae Species

**Figure 3:**
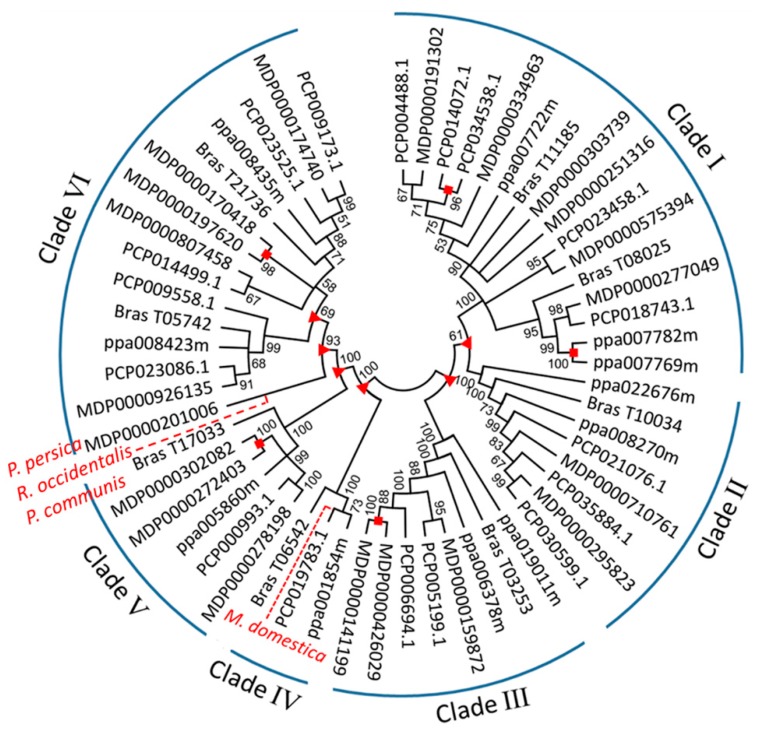
Phylogenetic tree analysis of *MDH* genes in Rosaceae genomes using NJ method. Old and recent duplication events are represented by red triangle and square, respectively. Gene loss is indicated by red dash line. Bootstrap values (higher than 50%) are shown near branched lines.

Four Rosaceae species, apple, peach, pear and black raspberry, were used to construct a phylogenetic tree, and phylogenetic analysis revealed that all the *MDH* genes were grouped into six clades (Figure 3). The bootstrap values of all the clades are greater than 50%, and the *MDH* genes from each Rosaceae species clearly separated in each clade. Of the 20 *MdMDH* genes, clades I, II, III, V and VI contained 6, 2, 3, 3, and 6 *MdMDH* genes, respectively. Furthermore, three sister pairs of *MdMDH* genes were identified in the phylogenetic tree, namely MDP0000426029–MDP0000141199 in clade III, MDP0000302082–MDP0000272403 in clade V, and MDP0000170418–MDP0000197620 in clade VI (Figure 3).

Depending on the time of occurrence, gene duplication can be divided into two types: old duplication and recent duplication [17]. If the gene duplication occurred within the species (species–species duplication), we considered a recent duplication; however, if the duplication event occurred prior to the radiation of Rosaceae species, we considered it an old duplication [18]. The signs of recent and old duplications within each *MDH* gene clade were detected in this study (Figure 3), and five incidences of recent duplication were identified, indicating that all the *MDH* genes, with the exception of five sister pairs of *MDH* genes, namely MDP0000426029–MDP0000141199, PCP014072.1–PCP034538.1, MDP0000302082–MDP0000272403, ppa007782m–ppa007769m, and MDP0000170418–MDP0000197620, existed before the Rosaceae species split. Furthermore, six incidences of old duplication were identified among the six tested clades, and two incidences of old duplication were identified in clade VI, indicating that the *MDH* gene clades underwent at least one duplication event prior to the Rosaceae species split. Recent gene loss in *M. domestica*, *P. communis*, *R. occidentalis* and *P. persica* was observed in clades IV and VI (Figure 3). However, signs of old loss of *MDH* genes were not detected.

### 2.4. Estimation of the Positive Selection of MDH Genes in Four Rosaceae Species

**Figure 4:**
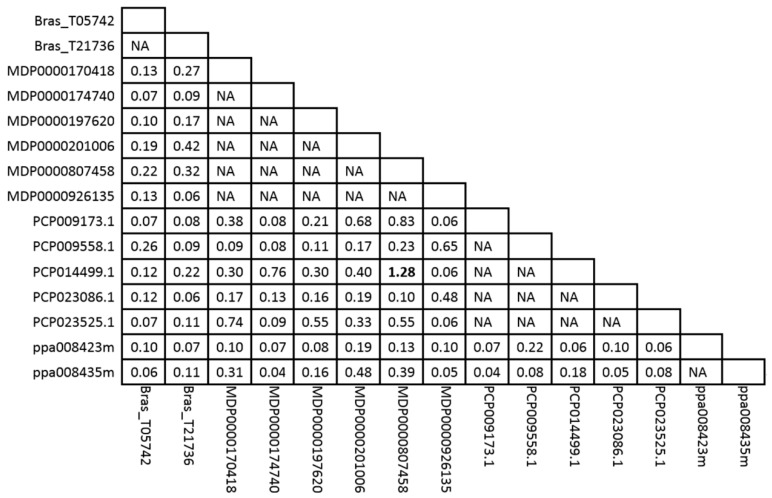
Estimation of ω values of *MDH* gene clade VI using the ML method. The ω value higher than one is highlighted in black bold.

To better understand whether the *MDH* genes in each clade are under different evolutionary constraints, the pairwise ω value for all the tested *MDH* genes was calculated using a pairwise comparison model (Figure 4 and Table S4). Furthermore, the pairwise ω values of *MDH* genes in each clade were compared, and the average pairwise ω values within each of the six clades followed the order of: clade IV > clade I > clade III > clade VI > clade II > clade V (Figure S1). In contrast, a significant difference in pairwise ω values was detected for *MDH* genes from clades IV and V (*p* < 0.01). In short, the pairwise ω values revealed that the evolutionary rates of the *MDH* genes in each clade differ. In addition, we compared the pairwise ω values within clade VI. Interestingly, positive selection was identified in one pair of orthologs (MDP0000807458 and PCP014499.1), with a ratio of 1.28. We then analyzed the genomic sequence of MDP0000807458 using 10 apple cultivars and 11 wild relatives (Table S5). A total of 53 single nucleotide polymorphisms (SNPs) in the promoter and coding sequences were identified among the tested samples. With respect to the 53 SNPs, 47 were located in the promoter, 3 were located in introns, and 3 were located in exons. However, the amino acid sequence of MDP0000807458 was no different between the apple cultivars and wild relatives, indicating that this gene clearly exhibited extreme conservation during apple domestication. Different *cis*-elements indicate various functions. In our study, a 1.8 kb long promoter region of MDP0000807458 was identified, and the Plant *Cis*-acting Regulatory DNA Elements (PLACE) website server (http://bioinformatics.psb.ugent.be/webtools/plantcare/html/) was used to analyze the promoter. A total of 17 *cis*-elements were identified in the promoter region of MDP0000807458 (Table 2). Of these *cis*-elements, four are responsive to light; three are responsive to plant hormones, such as auxin and methyl jasmonate (MeJA); and one is the MYB binding site involved in flavonoid biosynthesis. In addition, four *cis*-elements, the TATA-box, MYB binding sits, As element, and CAAT-box, were altered because of SNPs (Table 2 and Table S5). Hence, we considered these four *cis*-elements to be potential candidate sites related to functional divergence.

To better understand the evolutionary rate of the *MDH* clades, which had divided from the most recent common ancestor, the ω values of the different *MDH* clades were calculated via the branch-specific model in PAML. The results are shown in Table 3. The two-ratio model is favored in two pairs of *MDH* clades, III and I + II, as well as IV and V + VI, both of which had a *p*-value less than 0.01, suggesting that the ω values of clades III and IV differed significantly from those of the clades I + II and V + VI, respectively. However, the one-ratio model is favored over the others, which had *p*-values greater than 0.05.

### 2.5. Expression Analysis of the MdMDH Genes in Different Tissues of Apple Plants

**Figure 5:**
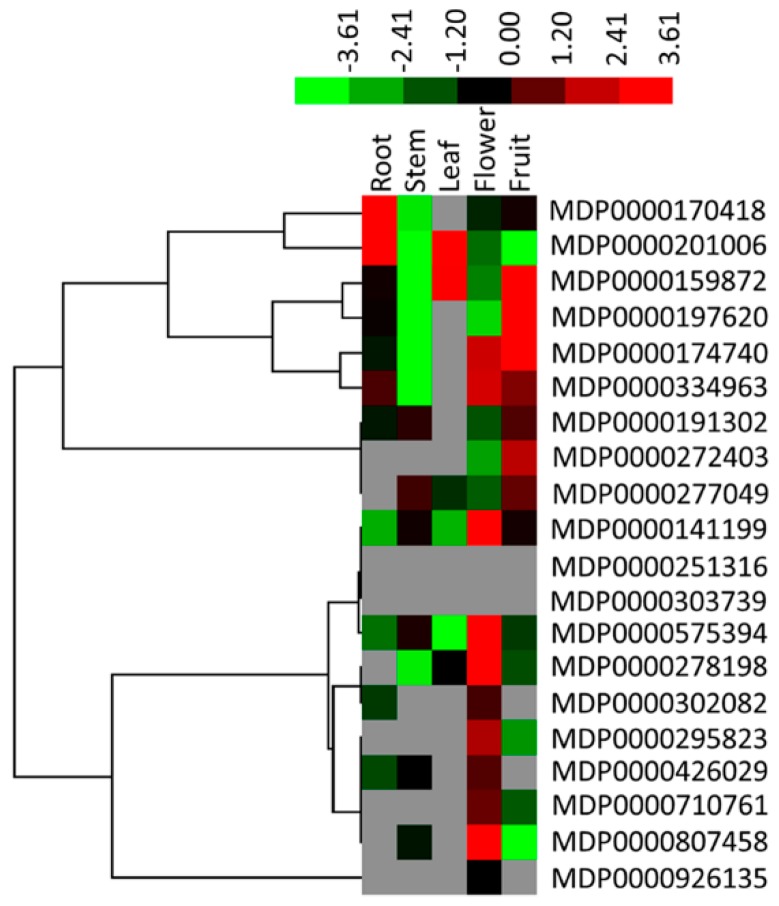
Expression profiles of *MdMDHs* in five tissues, including fully bloomed flowers (Flower), Root, mature leaf (Leaf), ripening fruit (Fruit) and Stem. The heat map was generated by Mev software based on relative expression levels of *MdMDHs*, and normalized log2 transformed values were used with hierarchical clustering. The different colors represent different expression level, with the red color representing the highest value of gene expression.

To better understand the tissue-specific expression patterns of the members of the *MDH* gene family in apple, qRT-PCR was used to elucidate the expression levels of 20 *MdMDH* genes in five type of tissue: root, stem, mature leaf, fully bloomed flower and ripening fruit tissue (Figure 5 and Table S6). The expression patterns of the *MdMDH* genes significantly differed among the different tissues, and the expression of two genes, MDP0000251316 and MDP0000303739, was not detected in any of the five tested tissue types. In addition, we found that nine *MdMDH* genes had a higher expression levels in the root, with the expression level above 0.1. Similarly, five *MdMDH* genes showed a higher expression levels in the stem, eleven *MdMDH* genes exhibited a higher expression in ripening fruits, four *MdMDH* genes had a higher expression levels in mature leaves. Twelve *MdMDH* genes showed a higher expression levels in full-blooming flowers, with the expression level above 1 (Figure 5). Furthermore, some *MdMDH* genes, such as MDP0000295823, MDP0000172403, MDP0000302082, MDP0000710761 and MDP0000926135, were expressed specifically in one or more tissues, indicating that these genes play an important role in apple specific tissues.

### 2.6. Expression Profiles of MdMDH Genes During Apple Flower Bud Differentiation and in Fruit at Different Developmental Stages

**Figure 6:**
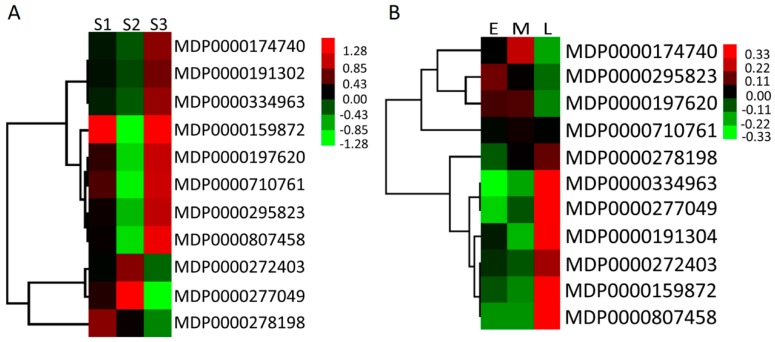
Expression level of *MdMDH* genes during apple fruit development (**A**) and flower bud differentiation (**B**) of apple cultivar “Qinguan”. The heat map was generated by Mev software based on RNA-seq database, and normalized log2 transformed values were used with hierarchical clustering. S1, S2 and S3 represent juvenile stage, expanding stage and mature stage of fruit development, respectively. E, M and L represent early stage, middle stage and late stage of flower bud differentiation, respectively.

**Figure 7:**
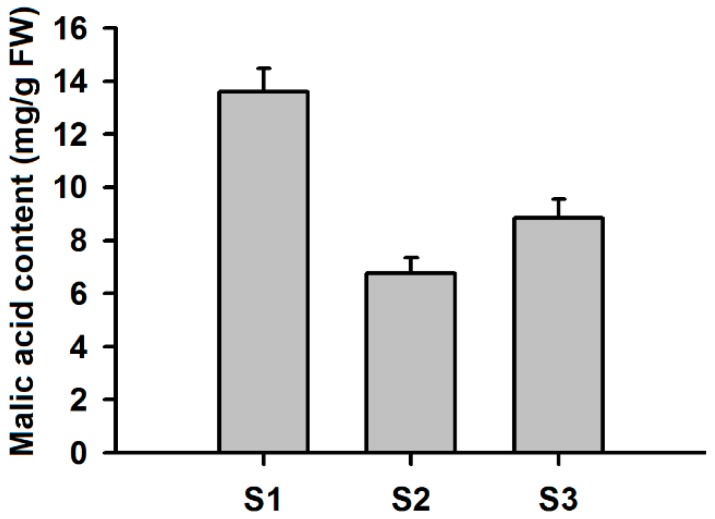
Changes in malic acid concentration in fruits of apple during development. S1, S2 and S3 represent juvenile stage, expanding stage and mature stage of fruit development, respectively. FW, fresh weight.

To further investigate the expression patterns of *MdMDH* genes during apple flower bud differentiation and in fruit at different developmental stages, six RNA-seq libraries, including three fruit and three flower RNA-seq libraries, were constructed and sequenced. RPKM values were used to estimate the expression levels of the *MdMDH* genes. Of these 20 *MdMDH* genes, 9 were not detected throughout fruit development and flower bud differentiation, and 11 genes were expressed during fruit development and flower bud differentiation (Figure 6). With respect to the fruits, the expression patterns of the *MdMDH* genes during fruit development were investigated, and the results showed that the expression of one gene (MDP0000278198) showed highly increased expression throughout fruit development, the expression of two genes (MDP0000272403 and MDP0000277049) was high at the expanding stage, and the expression of the other eight genes was high at the juvenile stage and mature stage of fruit development (Table S7). Malic acid content is one of the main determinants of fruit organoleptic quality. To better understand the relationship between *MdMDH* genes and malic acid content, the malic acid content was also determined via HPLC, and the results are shown in Figure 7. The average malic acid content was highest at the juvenile stage (13.58 mg/g FW) but decreased significantly at the expanding stage (6.76 mg/g FW), after which it increased slightly during the last stage of fruit development (8.86 mg/g FW). Among 11 highly expressed *MdMDH* genes throughout fruit development, 8 *MdMDH* genes show similar expression pattern to the pattern of malic acid accumulation. Thus, we considered that the *MdMDH* genes are involved in malic acid metabolism. Furthermore, we investigated the expression patterns of the *MdMDH* genes during flower bud differentiation of the apple cultivar “Qinguan” (Table S8). The expression levels of four genes (MDP0000272403, MDP0000278198, MDP0000710761 and MDP0000807458) were similar throughout flower bud differentiation. The expression levels of two genes (MDP0000334963 and MDP0000277049) strongly increased; however, the expression of MDP0000295823 decreased during flower bud differentiation. In addition, the expression of two genes (MDP0000174740 and MDP0000197620) was high at the middle stage of flower bud differentiation; however, the expression of two other genes (MDP0000191302 and MDP0000159872) was high during the late stage of flower bud differentiation. In short, the above results revealed that *MdMDH* genes are involved in organic acid metabolism and flower bud differentiation; however, the mechanisms governing the involvement of different *MdMDH* genes in organic acid metabolism and flower bud differentiation are complex and diverse.

## 3. Discussion

It is well known that *MDH* genes play crucial roles in energy homeostasis, plant development and cold and salt tolerance, as they mediate the reversible conversion of malate to oxaloacetate. In this study, members of the *MDH* gene family were first reported in apple based on genome-wide identification, together with their physiological and biochemical properties, chromosomal localization, gene duplication status, evolutionary rate, selective pattern and expression analysis.

The domesticated apple is diploid, has an autopolyploidy origin, and has a basic chromosome number of x = 17 [12,19]. Gene duplication is a major driving force for recruitment of genes in plants. During the process of apple domestication, genome-wide/segmental duplication and random duplication occurred widely within the apple genome [12,16,19]. In the present study, a total of 20 *MdMDH* genes were identified, 17 of which were located on six homologous pairs of chromosomes (Figure 2). For instance, chromosomes 3 and 11 are homologous pairs, and both contain a *MdMDH* gene at the bottom of each chromosome. Similar findings were also observed for homologous pairs 5–10, 2–7, 6–14, and 8–15. These results clearly indicated that the duplication of *MdMDH* genes is related to apple WGD. Chromosomes 6 and 14 are homologous pairs of chromosomes. Chromosome 6 contains one *MdMDH* gene (MDP0000159872) at the bottom, while two *MdMDH* genes (MDP0000426029 and MDP0000141199) were discovered within a homologous region on chromosome 14. In addition, we detected three sister pairs of *MdMDH* genes, MDP0000426029–MDP0000141199, MDP0000302082–MDP0000272403 and MDP0000170418–MDP0000197620, which were generated by recent duplication event (Figure 2 and Figure 3). These results indicate that tandem duplication of *MdMDH* genes likely occurred on chromosomes 14, 16 and 17. Notably, a single *MdMDH* gene was detected at the top chromosome of 10, while no *MdMDH* gene was detected on chromosome 5, which is homologous to chromosome 10. Similar results were also observed for chromosomes 7, 13 and 16. Because duplicated gene copies after WGD exhibited rapid gene divergence and loss [20], we speculate that the *MdMDH* genes on chromosomes 7, 13 and 16 were lost in the ancestor of apple. In addition, one *MdMDH* gene (MDP0000303739) was identified among the unanchored sequences, and phylogenetic analysis revealed that this gene is closely related to MDP0000251316 (Figure 3). Thus, we speculate that the *MdMDH* gene (MDP0000303739) among the unanchored sequence is likely to be located at the top of chromosome 5.

In the present study, a total of six clades were identified based on the amino acid sequences of *MDH* genes from four Rosaceae species (Figure 3). Based on the phylogenetic tree, the *MdMDH* genes were grouped with the *MDH* genes from pear, indicating a close relationship between *MDH* genes from apple and pear. There are 20, 17, 11 and 8 *MDH* genes in apple, pear, peach and black raspberry, respectively. There are nearly twice the number of *MDH* genes in apple and pear as there are in peach and black raspberry (Figure 3). In addition, the number of haploid chromosomes of apple and pear is 17 compared with 8 in peach and 7 in black raspberry. Thus, we presume that apple and pear have experienced a WGD event, which is consistent with previous reports [12,21]. However, peach and black raspberry have not experienced this type of event. Apple is a diploidized autopolyploid species, and a portion of polyploidy-derived duplicated genes has been retained under positive selection, whereas others have been lost during evolution and speciation [22].

Gene duplication is considered as the predominant influencing factor of adaptive functional novelty in plants [13,15], and the expression of most duplicated genes is highly divergent [14]. In the present study, this was also the case for the *MdMDH* genes. For instance, the expression pattern of two *MdMDH* homologous genes, MDP0000277049 and MDP0000926135, which are located on the homologous pairs of chromosomes 5 and 10, respectively, diverged (Figure 5 and Table S6). MDP0000277049 was highly expressed in stems, mature leaves, fully bloomed flowers and mature fruits; however, MDP0000926135 was highly expressed only in fully bloomed flowers. In addition, two *MdMDH* genes, MDP000710761 and MDP0000295823, which are located on the homologous pairs of chromosomes 3 and 11, respectively, showed similar expression patterns. These two *MdMDH* genes were highly expressed in fully bloomed flowers and mature fruits; however, the expression level of MDP000710761 was lower than that of MDP0000295823. MDP0000426029 and MDP0000141199 are clustered at the bottom of chromosome 11. The expression of MDP0000144199 was low in five tested apple tissue types, but the expression of MDP0000426029 was high in the stems, roots and fully bloomed flowers. Similarly, MDP0000272403 and MDP0000302082 are clustered at the top chromosome 16. MDP0000272403 was highly expressed in fully bloomed flowers and mature fruits; however, MDP0000302082 was high expressed in roots and fully bloomed flowers. Thus, we considered that the expression of the duplicated *MdMDH* genes derived from GWD/segmental duplication or tandem duplication has diverged.

The formation of flower buds and fruits on apple trees plays an important role in the life cycle of trees and is regulated by complex networks. In this study, a total of 11 *MdMDH* genes were found to be expressed during fruit development and flower bud differentiation. Among these 11 *MdMDH* genes, one gene, MDP0000807458, was identified to be under positive selection during apple domestication (Figure 4). In addition, the expression level of MDP0000807458 was highest in fully bloomed flowers, and the expression of MDP0000807458 was similar throughout flower bud differentiation. Thus, we speculate that MDP0000807458 is likely candidate gene involved in the regulation of flower bud differentiation It is worth clarifying the function of MDP0000807458 in the future.

## 4. Materials and Methods

### 4.1. Identification of MDHs and Phylogenetic Analyses

The amino acid sequences of the *MDH* genes in *A. thaliana* [6] were downloaded from The Arabidopsis Information Resource (TAIR) (https://www.arabidopsis.org/), and used as query sequences to identify homologous genes in the reference genome sequence of apple (*M. domestica*) [12], pear (*Pyrus communis*) [23], peach (*Prunus persica*) [24] and black raspberry (*Rubus occidentalis*) [25] via the Basic Local Alignment Search Tool (BLAST) according to the procedures described by Tatusov et al. [26] and Wang et al. [18]. In brief, BLAST 2.2.24 software (http://blast.ncbi.nlm.nih.gov/Blast.cgi) was used to construct a local BLAST database for each species, and the putative orthologous genes were identified using all-against-all protein BLAST in conjunction with reciprocal best similarity matching. These analyses were performed to identify homologous genes.

With respect to the phylogenetic analysis, multiple alignments of the amino acid sequences were performed using ClustalX2, and the resulting data were used to construct a phylogenetic tree via the MEGA 7 software (https://www.megasoftware.net/) with both the maximum likelihood (ML) and neighbor joining (NJ) methods [27]. With respect to the NJ method, the following parameters were used: bootstrap, 1000 replicates; *p*-distance; and pairwise deletion. Regarding the ML method, the following parameters were used: bootstrap, 1000 replicates; Jones–Taylor–Thornton; partial deletion; and branch swap filter, very strong.

### 4.2. Gene Structure and Conserved Motif Analysis of MDH Genes

The structural features of *MdMDH* genes were displayed via Gene Structure Display Server 2.0 (http://gsds.cbi.pku.edu.cn/) based on the alignment of their coding sequences with their corresponding genomic sequences. The MEME suite server (http://meme-suite.org/) was used to identify the conserved motifs of the *MdMDH* genes, and the parameters used in this study were as follows: maximum number of different motifs, 10; minimum width, 10; and maximum width, 50.

### 4.3. Estimations of Gene Duplication and Gene Loss

Based on the phylogenetic analysis, gene duplication and gene loss were estimated by manually checking each clade. It was assumed that each gene clade comprises at least one *MDH* gene member from each species. If there were two or more clades, or two or more members of each species, the gene was considered to have experienced one or more gene duplications. Moreover, it was considered a gene loss if a *MDH* gene member was not found in one or more species of each clade. Each identified gene loss was further confirmed by searching the GenBank database via BLASTP.

### 4.4. Positive Selection Analysis

Clustal X2 was used to align the amino acid sequences, and multiple alignments of the proteins and their corresponding coding sequences were submitted to PAL2NAL (http://www.bork.embl.de/pal2nal/). The output result was used to estimate the ω values between the different branches of the phylogenetic tree using a branch-specific algorithm in Codeml from PAML 4.9 (http://abacus.gene.ucl.ac.uk/software/paml.html) [28]. In brief, a constant ω value (dN/dS ratio) for all branches is assumed in the one-ratio model, while the ω values (dN/dS ratio) are assumed to vary for different branches (the background and the foreground branches) in the two-ratio model. Two times the log-likelihood difference (2∆ln*L* = 2(ln*L*_1_ − ln*L*_0_) was used to test for significant differences between two models, and the optimized ω values were determined in both models using the likelihood ratio test (LRT).

The coding sequence of the *MDH* genes were aligned using Clustal X2. The resultant output data were used to calculate the ω value (*Ka*/*Ks* ratio) using the *KaKs* calculator package 2.0 (https://sourceforge.net/projects/kakscalculator2/) in conjunction with the ML method [29]. A pairwise comparison was used to test the variation of the ω values of the *MDH* genes among the different clades of the phylogenetic trees. Briefly, the ω values between two randomly selected sequences within each clade were calculated using the *KaKs* calculator. Statistical analysis was carried out using SPSS statistics 17.0 software (SPSS Inc., Chicago, IL, USA), and t-tests were used for significant differences in the ω values of *MDH* genes between the different clades.

### 4.5. RNA Isolation and Quantitative RT-PCR (qRT-PCR) Analysis

Five types of apple tissue including fully bloomed flower, root, mature leaf, ripening fruit and stem tissue, were used for qRT-PCR assays. A Wolact Plant RNA Isolation Kit (Wolact, Hong Kong, China) was used to extract the total RNA according to the manufacturer’s instructions, and first-strand cDNA was synthesized using TransScript One-Step gDNA Removal and cDNA Synthesis SuperMix (Trans, Beijing, China) in accordance with the manufacturer’s instructions. qRT-PCR with SYBR Green I Master Mix (TaKaRa, Dalian, China) was performed on an Applied Biosystems 7500 Real-Time PCR System (Applied Biosystems, Foster City, CA, USA). The relative expression level for each gene was measured according to the cycle threshold (*Ct*), which is also referred to as the 2^−ΔΔ*C*T^ method, and all analyses included three biological replicates. An actin gene described in a previous study was selected as a constitutive control [30]. All primers used for qRT-PCR are listed in Table S1.

### 4.6. Plant Material and Transcriptome Analysis

The apple cultivar “Qinguan” and an individual plant from a segregating F1 apple population derived from a cross between “Qinguan” and “Honeycrisp” used in this study were maintained at the Horticultural Experimental Station of Northwest A&F University, Yangling, Shaanxi Province, China. Fruit samples were collected at three stages, the juvenile stage, expanding stage, and mature stage, which corresponded to 30, 60 and 90 days after full bloom (DAFB), respectively. All fruit samples were peeled, after which the pulps were cut into small sections, immediately frozen in liquid nitrogen, and then stored at −80 °C for transcriptome sequencing and organic acid content measurements. In addition, buds sampled during bud growth were collected at the early stage, middle stage and late stage of flower bud differentiation according to the methods described by Xing et al. [31,32], directly frozen in liquid nitrogen, and then stored at −80 °C for transcriptome sequencing. An RNA sequencing (RNA-seq) library was constructed by the Biomarker Biotechnology Corporation (Beijing, China) in accordance with the methods of Lou et al. [33]. Transcriptome sequencing was conducted on an Illumina HiSeq™ 2000 platform (Illumina, San Diego, CA, USA). The reads per kilobase per million reads (RPKM) value was used to estimate the gene expression variation. The RNAseq data of flower bud differentiation of apple cultivar “Qinguan” were uploaded to the public open data in NCBI Sequence Read Archive by Libo Xing (https://trace.ncbi.nlm.nih.gov/Traces/sra_sub/).

### 4.7. Measurements of Malic Acid Contents in Apple Fruits

The malic acid contents in the apple fruits were measured using High Performance Liquid Chromatography (HPLC) as described in our previous protocol [34]. Briefly, approximately 5 g of frozen powder was dissolved in 20 mL of ddH_2_O, which was generated by a Milli-Q Element water purification system (Millipore, Bed ford, MA, USA). The mixture was put into an ultrasonic bath and maintained at room temperature for 30 min under continuous ultrasonic shaking. The mixture was then centrifuged at 1200 rpm for 15 min at 4 °C The supernatants were decanted and filtered through a 0.22 μm Sep-Pak filter (Anpel, Shanghai, China). The filtered fluid was used to measure the malic acid content via an Agilent 1260 Infinity HPLC system (Milford, MA, USA) coupled to a diode array detector at 210 nm. Chromatographic separation was performed on an Athena C18 column (1000 nm, 4.6 nm × 250 nm, 5 μm); the column temperature was maintained at 40 °C The mobile phase was a 0.02 mol/L KH_2_PO_4_ solution at a pH of 2.4, and the flow rate was 0.8 mL/min. Three biological replicates were conducted. The malic acid standard used was purchased from Sigma (St. Louis, MO, USA), and dissolved in deionized water.

## 5. Conclusions

To our knowledge, this present study provides a deep understanding of the evolutionary pattern of *MDH* genes in apples systematically. Overall, a total of 20 *MdMDH* genes were identified in the “Golden Delicious” apple draft genome. The numbers of exons and conserved motifs within the *MdMDH* genes ranged dramatically, indicating extreme divergence of the *MdMDH* genes during the evolutionary process. The chromosomal localization analysis of the *MdMDH* genes revealed that GWD, segmental duplication, tandem duplication and gene loss occurred during apple domestication. The *Ka*/*Ks* ratio of the duplicated *MdMDH* genes indicated that different selective pressures acted on the *MdMDH* genes during apple domestication.

A phylogenetic tree was constructed based on the amino acid sequences of *MDH* genes from four Rosaceae species, and six clades were identified. Based on the phylogenetic relationships, five incidences of recent duplication, six incidences of old duplication and significant differences in the evolutionary rates of members of the *MDH* gene family were identified. In addition, significant divergence in expression profiles of *MdMDH* genes among five types of apple tissue was observed. Eleven *MdMDH* genes were highly expressed during fruits development and flower bud differentiation. One gene, MDP0000807458, was highest expressed in fully bloomed flowers and identified to be under positive selection during apple domestication. In addition, the expression of MDP0000807458 was similar throughout flower bud differentiation, Thus, our results suggest that MDP0000807458 is a likely candidate gene involved in the regulation of flower bud differentiation.

## Figures and Tables

**Table 1:** Summary information of physiological and biochemical properties of the MdMDH proteins.

Gene ID	Animo acids	MW (kDa)	pI	GRAVY	Instability index	Aliphatic index
MDP0000159872	413	43.57	8.10	0.052	34.70	94.77
MDP0000426029	413	43.44	6.61	0.076	33.78	94.77
MDP0000334963	361	38.12	8.46	0.136	25.09	98.59
MDP0000710761	339	35.51	8.98	0.120	43.04	101.27
MDP0000277049	397	41.76	7.57	0.231	24.20	101.89
MDP0000303739	360	38.47	8.86	0.133	30.27	101.25
MDP0000191302	362	38.94	8.84	0.204	33.03	105.50
MDP0000295823	946	103.76	8.32	−0.085	46.76	95.25
MDP0000141199	224	23.49	8.93	0.087	32.91	88.08
MDP0000251316	289	31.21	8.86	−0.030	34.54	99.07
MDP0000575394	283	30.95	6.02	0.008	43.55	92.93
MDP0000174740	465	50.03	6.38	0.010	30.12	97.12
MDP0000926135	376	41.26	7.05	0.062	30.35	102.42
MDP0000197620	468	51.69	7.02	−0.050	36.72	95.00
MDP0000170418	542	60.45	8.80	−0.044	43.04	93.87
MDP0000272403	478	52.82	5.99	−0.058	31.05	94.87
MDP0000302082	489	54.12	6.09	−0.048	31.15	96.32
MDP0000278198	517	57.10	6.05	−0.029	29.90	97.35
MDP0000807458	134	14.48	9.27	0.172	32.91	99.63
MDP0000201006	154	16.87	9.22	−0.019	34.47	99.35

MW, Molecular weight of the amino acid sequence; pI, theoretical isoelectric point; GRAVY, grand average of hydropathicity.

**Table 2:** Identification of putative cis element in the promoter of MDP0000807458 gene.

No.	Cis-Element Name	Cis-Element Sequence	Function
1	AE-box	AGAAACAA	Light responsive element
2	ARE	AAACCA	regulatory element essential for the anaerobic induction
3	As	TGACG	unknown
4	CAAT-box	CCAAT	cis-acting element in promoter and enhancer regions
5	CGTCA-motif	CGTCA	MeJA-responsiveness
6	CTAG-motif	ACTAGCAGAA	unknown
7	GA-motif	ATAGATAA	Light responsive element
8	GATA-motif	AAGATAAGATT	Light responsive element
9	GT1-motif	GGTTAA	Light responsive element
10	MBSI	AAAAAACSGTTA	MYB binding site involved in flavonoid biosynthesis
11	MYB	TAACYR	unknown
12	MYC	TCTCTTA/CAATTG	unknown
13	STRE	AGGGG	unknown
14	TATA-box	TATWWAAW	unknown
15	TGACG-motif	TGACG	MeJA-responsiveness
16	TGA-element	AACGAC	auxin-responsive element
17	WUN-motif	AAATTTCCT	wound-responsive element

**Table 3:** Likelihood-ratio test (LRT) statistic and parameters from Branch model of PAML.

Clade	Null Hypothesis		Alternative Hypothesis			LRT	
	−In L	ω	−In L	ω1	ω2	Statistic	*p*
I+II	1777.25	0.12	1776.71	0.11	0.08	1.08	> 0.05
(I + II) + III	2305.32	0.15	2299.96	0.10	0.28	10.7	< 0.01
(V + VI) + IV	1991.21	0.13	1987.85	0.12	0.55	6.7	< 0.01
V + VI	1555.13	0.12	1553.69	0.13	0.07	2.9	> 0.05

## Supplementary Materials

Supplementary materials can be found at http://www.mdpi.com/1422-0067/19/11/3312/s1.
