# Supplementary figures and images for: Genome-wide Identification, Classification, Molecular Evolution and Expression Analysis of Malate Dehydrogenases in Apple
Source: Int J Mol Sci. 2018 Oct 24;19(11):3312. doi: 10.3390/ijms19113312 (PMC6274877; doi:10.3390/ijms19113312)

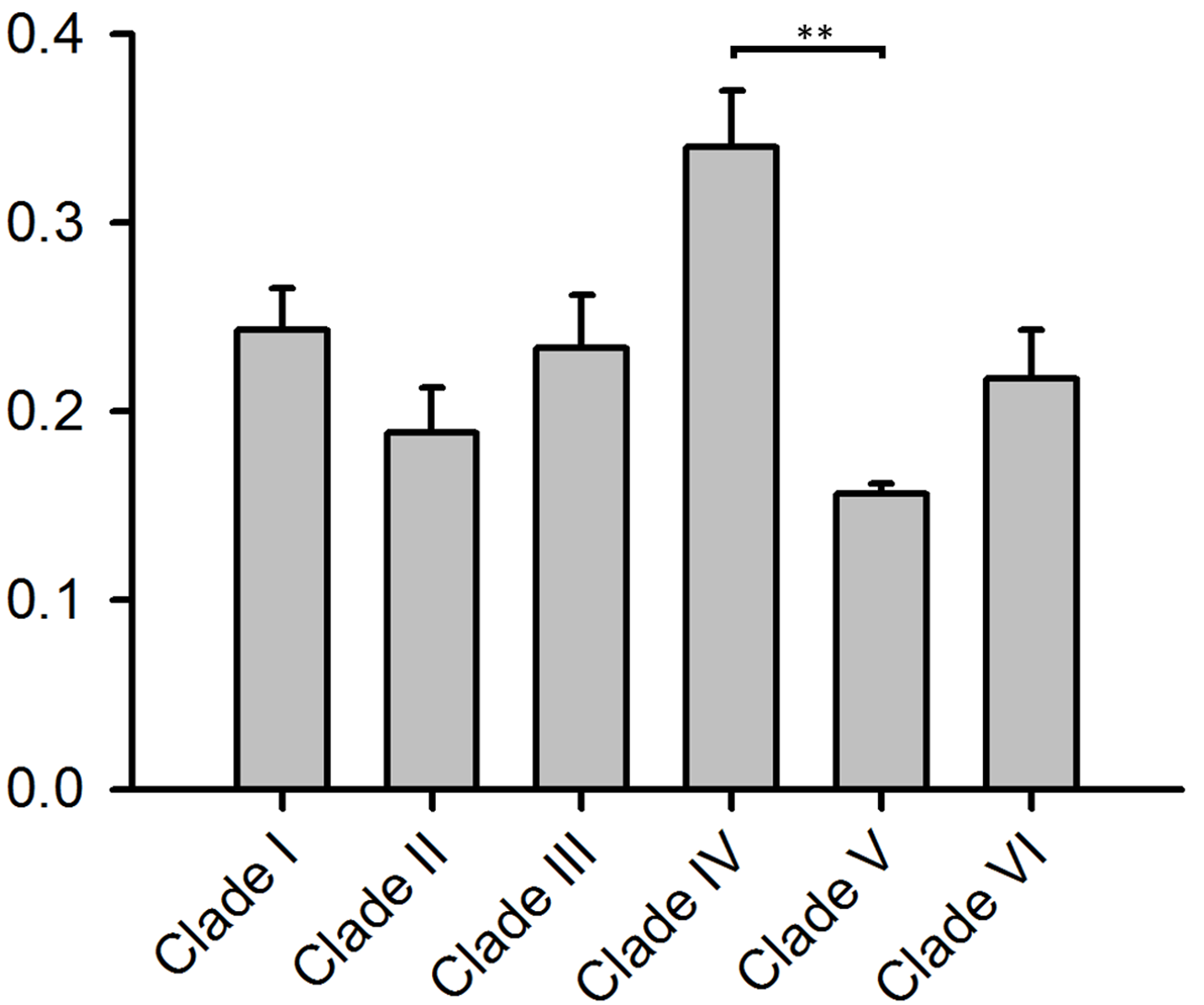

Supplement: Supplementary file 1 [file ijms-19-03312-s001.zip › supplemental materials/Fig.S1 .TIF]
